# HPV Infection in Children and Adolescents—A Comprehensive Review

**DOI:** 10.3390/jcm14072425

**Published:** 2025-04-02

**Authors:** Paulina Tomecka, Anna Karwowska, Jacek Kuźnicki, Katarzyna Skinderowicz, Aleksandra Wojno, Kornelia Markut, Paulina Typek, Urszula Ciesielska, Julita Kulbacka, Małgorzata Drąg-Zalesińska

**Affiliations:** 1 Faculty of Medicine, Wroclaw Medical University, 50-367 Wroclaw, Poland; paulina.tomecka@student.umw.edu.pl (P.T.); anna.karwowska@student.umw.edu.pl (A.K.); jacek.kuznicki@student.umw.edu.pl (J.K.); katarzyna.skinderowicz@student.umw.edu.pl (K.S.); aleksandra.wojno@student.umw.edu.pl (A.W.); kornelia.markut@student.umw.edu.pl (K.M.); paulita.typek@student.umw.edu.pl (P.T.); 2 Department of Human Morphology and Embryology, Division of Histology and Embryology, Faculty of Medicine, Wroclaw Medical University, T. Chalubińskiego 6a, 50-368 Wroclaw, Poland; urszula.ciesielska@umw.edu.pl; 3 Department of Molecular and Cellular Biology, Faculty of Pharmacy, Wroclaw Medical University, Borowska 211a, 50-556 Wroclaw, Poland; 4 Department of Immunology and Bioelectrochemistry, State Research Institute Centre for Innovative Medicine, Santariškių g. 5, LT-08406 Vilnius, Lithuania

**Keywords:** human papillomavirus (HPV), paediatric infections, vertical transmission, HPV DNA prevalence, maternal–child transmission, HPV-related morbidity

## Abstract

**Background:** Human Papillomavirus (HPV) is a predominant and clinically significant virus affecting individuals of all ages, including children and adolescents. Despite its well-documented role in adult health, particularly in cervical cancer, HPV’s impact on younger populations still remains underexplored. **Methods:** This review investigates the epidemiology, clinical manifestations, transmission pathways, and historical context of HPV in children and adolescents. **Results:** The study demonstrates a significant prevalence of HPV DNA within paediatric populations, with diverse clinical manifestations such as verruca vulgaris, anogenital warts, and Juvenile Recurrent Respiratory Papillomatosis, alongside substantiating vertical transmission from mother to infant. We also highlight ground-breaking research milestones, including improvements in genetic studies, the development of HPV vaccines, and ongoing investigations into infection dynamics, and long-term health outcomes. **Conclusions:** By synthesising existing knowledge, this review aims to enhance clinical decision-making, improve management strategies, and pave the way for future research in HPV-related paediatric diseases.

## 1. Introduction

Human Papillomavirus (HPV) is a widespread and clinically significant virus that affects both adults and children, representing a major public health concern globally [1]. The distribution and burden of HPV infections vary widely across different populations and regions, influenced by factors such as geography, socio-economic conditions, cultural practices, genetic predisposition, and individual characteristics like age, sex, infection site, and overall health [2].

The recognition of HPV infection in children dates back to ancient Greek and Roman medical observations of skin and genital warts [3]. A pivotal study by Pfister and Zur Hausen in 1978 advanced understanding by using molecular hybridisation techniques to analyse HPV in paediatric cases [4]. Their findings revealed that certain HPV types (1–3) predominantly affect children aged 5 to 15 years, laying the foundation for further studies on HPV’s impact in paediatric dermatology and oncology [5].

While the impact of HPV is well-documented in adults, particularly in relation to cervical cancer, its prevalence and implications in paediatric populations are less understood and require further investigation [6,7,8].

HPV infection in children can present as common skin warts, anogenital warts, or, in rare cases, Juvenile Recurrent Respiratory Papillomatosis (RRP), which is particularly concerning [9]. Warts are rare in infancy and early childhood, with their prevalence rising during school age and peaking between 12 and 16 years [10]. Exposure to HPV within family and school environments significantly contributes to wart development in children, with higher transmission risks in these settings [11]. The prevalence of HPV DNA among children aged 1 month to 4 years ranges from 55% to 70%, comparable to the 75% prevalence observed in their parents and other adults, as reported in 2003 research [12]. A study conducted in the Netherlands, published in 2009, involving 1465 children aged 4–12 found that 33% had skin warts. Among these children, 9% exhibited palmar warts, 20% had plantar warts, and 4% had both types, emphasising the significant prevalence and the importance of effective management strategies [13].

Although rare, anogenital warts in children have been observed in regions such as the labia, vagina, urethra, and perianal area, with girls being more frequently affected [14,15]. A pilot study conducted from 2012 to 2014 involving 95 participants aged 2 to 21 revealed a genital HPV infection prevalence of 37.9%, including both sexually active (47.4%) and inactive adolescents (28.6%), as well as prepubescent children (34.5%) [16]. Newborns can also acquire HPV, with HPV DNA detected in oral swabs ranging between 4% and 87% [17]. In a Polish study from 2014, HPV was identified in 22% of pregnant women, and 53.3% of their newborns were also found to be infected [18].

This review reveals a notable prevalence of HPV DNA in paediatric populations, with a variety of clinical manifestations, including verruca vulgaris, anogenital warts, and RRP, while also supporting evidence of vertical transmission from mother to child. Additionally, we emphasise key advancements in research, such as progress in genetic analyses, the development of HPV vaccines, and ongoing efforts to understand the infection’s dynamics and its long-term health impacts. By reviewing current findings, this article seeks to inform clinical decision-making, optimise management approaches, and contribute to the advancement of future research on HPV-related diseases in children.

## 2. Virus Characterisation

There are over 200 distinct HPV genotypes, classified into five major genera: Alpha, Beta, Gamma, Mu, and Nu papillomaviruses [19,20]. HPV exhibits significant genomic diversity, influencing its ability to infect various tissues and cause a range of clinical pathologies, including benign warts, genital lesions, and cancers [21]. Alphapapillomaviruses primarily infect the mucosal epithelium and are classified into high-risk and low-risk types based on their oncogenic potential [22]. Mucosal HPV infections are most commonly acquired during the initial stages of sexual activity in adulthood, although non-sexual transmission is also possible [23]. The International Agency for Research on Cancer (IARC) has classified twelve HPV types (16, 18, 31, 33, 35, 39, 45, 51, 52, 56, 58, and 59) as carcinogenic, particularly in relation to cervical cancer [24]. HPV infections are linked to various cancers, including cervical, anal, and genital cancers (vulvar, vaginal, penile), as well as head and neck cancers (HNCs) [25]. Persistent infection with high-risk HPV types and the aberrant expression of viral genes are key factors in the pathogenesis of HPV-associated carcinogenesis [26]. It is thought that the persistence of HPV infection and the progression to cervical cancer may be influenced by several other risk factors, such as early sexual debut, having multiple sexual partners, smoking, and the presence of other sexually transmitted infections [27]. Low-risk HPV types are capable of infecting both mucosal and cutaneous tissues, while all high-risk HPV types infect and replicate exclusively in the mucosal epithelium [28]. Lesions caused by low-risk HPV are typically self-limiting and resolve naturally through the host immune response. However, in susceptible populations, low-risk mucosal HPV infections can be difficult to manage and may lead to complications such as RRP [29]. Cutaneous HPV types, primarily from the Beta and Gamma genera, infect the squamous epithelium of the skin and are responsible for common, plantar, and flat warts, typically appearing on the hands, feet, and face [23,30]. These infections are primarily transmitted through skin-to-skin contact, especially among young children [23]. Beta HPV types are a natural component of the skin microbiota; however, under conditions such as immunosuppression or excessive UV exposure, they have been implicated in cutaneous carcinogenesis, though their precise role remains under investigation [31,32]. HPV 5 and HPV 8, both belonging to Beta HPV types, were first identified in patients with epidermodysplasia verruciformis, which increases susceptibility to Beta HPV infections and non-melanoma skin cancer [33].

### The HPV Genome and Proteins

HPV particles are spherical, 50 to 55 nanometres in diameter, with a capsid made of 72 identical icosahedral subunits that enclose its genome [34]. The HPV genome is a circular double-stranded DNA molecule that contains eight open reading frames (ORFs) encoding various viral proteins [35]. These ORFs are categorised into three primary regions: the Long Control Region (LCR), the early (E) region, and the late (L) region. The LCR is a non-coding segment of 400 to 1000 base pairs responsible for regulating transcription and replication. The E region encodes regulatory proteins, while the L region encodes structural proteins, including capsid proteins L1 and L2 [34]. L1, which accounts for about 80% of the viral capsid proteins, is the most conserved protein among papillomaviruses. L2, though a minor component, is crucial for packaging the viral genome within the capsid [35]. Additional key viral proteins include E1 and E2, which regulate DNA transcription and replication. Proteins E4, E5, E6, and E7 are involved in disrupting the cell cycle, evading the host immune system, and recruiting host replication factors [36]. The sustained proliferation of HPV-positive cancer cells depends on the continued expression of the viral oncogenes E6 and E7. These oncogenes interact with several cellular mechanisms, although the complete details remain to be fully understood [37].

**Figure 1:**
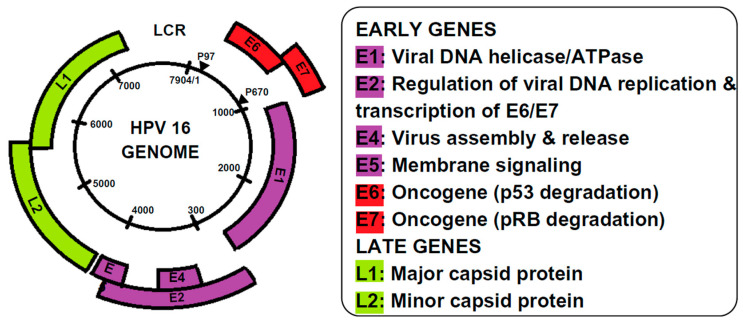
Genomic organisation of HPV 16.

HPV gene expression is polycistronic and regulated by multiple promoters [38]. For instance, in HPV-16, the two main promoters, p97 and p670, are crucial for viral function [39] (Figure 1). Along with host and environmental factors, the HPV genotype influences the type and malignant potential of the lesions [40].

## 3. Routes of Infection

**Figure 2:**
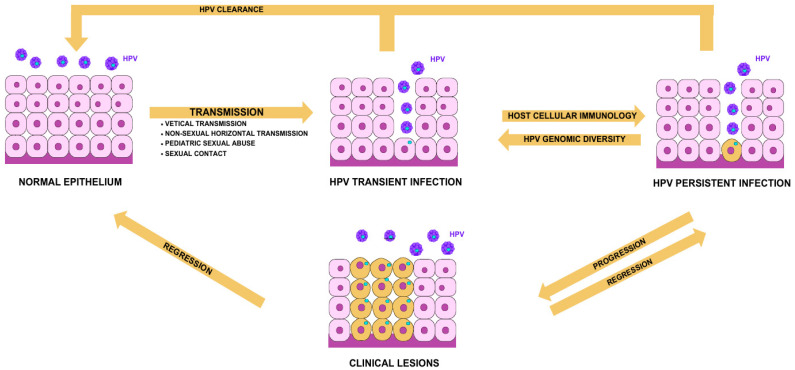
Transmission pathways and clinical outcomes of HPV in neonates and children. HPV is primarily transmitted through sexual contact, but alternative routes of transmission are particularly significant in the paediatric population. These routes are categorised into three main groups: vertical transmission, non-sexual horizontal transmission, and paediatric sexual abuse. This transmission may initially cause a transient HPV infection, which can either resolve spontaneously or progress to a persistent infection. Persistent HPV may either regress on its own or become symptomatic, leading to clinical lesions in various anatomical locations. In neonates and children, HPV often clears naturally.

Most HPV infections are acquired through sexual contact; however, clinical data support alternate routes of transmission, particularly in relation to the paediatric population [41]. Other modes of transmission are typically categorised into three main groups: vertical transmission, non-sexual horizontal transmission, and paediatric sexual abuse (Figure 2) [7].

**Figure 3:**
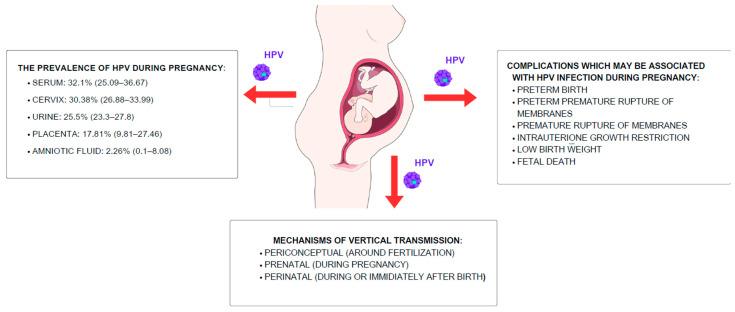
Manifestations and transmission pathways of HPV during pregnancy. Research into vertical transmission pathways focuses on HPV prevalence during pregnancy. HPV is generally more prevalent in pregnant women due to immune and hormonal changes, which may increase lesion severity. HPV DNA has been detected in samples such as the cervix, serum, urine, placenta, and amniotic fluid, with prevalence varying by sample type. Vertical transmission can occur through three mechanisms: periconceptual (around fertilisation), prenatal (during pregnancy), and perinatal (during or immediately after birth). The virus may be passed from mother to baby through ascending genital tract infections, transplacental transmission, or potentially via the bloodstream. HPV infection has been associated with complications such as preterm birth, membrane rupture, intrauterine growth restriction, low birth weight, and foetal death. This schematic was created using Servier Medical Art templates, which are licensed under CC BY 4.0; https://smart.servier.com (accessed on 20 August 2024).

Research into pathways of vertical transmission involves assessing HPV prevalence in pregnancy (Figure 3). Generally, HPV is more prevalent in pregnant women compared to non-pregnant ones due to immune and hormonal changes that can additionally lead to more severe manifestations of the virus, such as an increasing number of lesions [26]. HPV DNA has been detected in samples derived from the cervix, serum, and urine, along with placenta and amniotic fluid, and the overall prevalence of HPV among pregnant women varies regarding the sampling site [42]. Furthermore, HPV infection may be linked to various complications of pregnancy, including preterm birth, preterm premature rupture of membranes, and the premature rupture of membranes, as well as intrauterine growth restriction, low birth weight, and foetal death [7].

Vertical transmission can theoretically occur through three mechanisms: periconceptual (around fertilisation), prenatal (during pregnancy), and perinatal (during or immediately after birth). HPV can be passed from the mother to the baby at different stages of the pregnancy as a result of ascending infection from the maternal genital tract followed by transplacental transmission or, possibly, through the haematogenous route [43]. This is supported by the findings of a 2013 study that tracked 153 pregnant women to detect HPV at different stages of pregnancy and at birth. In total, 24% of the women had HPV at some point during their pregnancy—14% of women in the first trimester, 18% in the second, and 10% in the third. At birth, 5.2% (8/153) of newborns were also found to have HPV, with seven out of eight cases linked to mothers who were HPV-positive during pregnancy [44].

Additionally, during delivery, direct contact between the foetus and the mother’s infected genital tract can lead to perinatal transmission. This theory may be supported by the fact that similar HPV types were found in maternal cervicovaginal samples and oral samples derived from neonates [7,45]. It is considered that the mode of delivery influences the risk of transmission. The findings show that vaginal delivery increases the risk of HPV in infants, while caesarean delivery reduces it, even when membranes are ruptured before caesarean section. These results suggest that HPV is mostly transmitted during passage through the vaginal canal. However, study results alone do not recommend routine caesarean sections for HPV-positive women [46]. This is consistent with the American College of Obstetricians and Gynecologists (ACOG) guidelines, according to which the decision regarding the mode of delivery should be individualised based on various factors [47].

Despite the above, some research indicates that perinatal transmission is less clinically significant. In a prospective study by Khayargoli et al., 40.3% of the 1050 pregnant women involved were HPV-positive, while 7.2% of newborns tested positive for the virus at birth or within the first three months. However, in this cohort, all neonatal infections cleared by six months, suggesting that the perinatal transmission of HPV is infrequent and transient [48]. As for the periconceptual mechanism, studies are generally lacking; however, HPV has been detected in semen, possibly affecting its quality. The research has primarily focused on the impact of HPV on both male and female fertility, suggesting that HPV-infected sperm can carry the virus’s DNA to the egg, consequently influencing the development of the embryo and its ability to implant in the uterus [49].

HPV can spread through non-sexual horizontal routes, including contact with fomites, fingers, the mouth, and skin. Studies highlight that HPV is a resilient virus, capable of surviving on surfaces, clothing, and medical equipment for days [50]. In the context of horizontal transmission, the role of epithelial tissue stem cells as potential targets for HPV infection should be highlighted. HPV can infect various epithelial sites, including stem cells located in the bulge region of hair follicles, in sweat glands, as well as dispersed stem cells between follicles. In addition, specialised epithelial sites like the salivary glands and tonsillar crypts are also vulnerable to HPV infection. The virus is thought to access these stem cells through wounds, hair follicles, or ducts [51].

Heteroinoculation primarily occurs within families, with transmission possible through activities such as kissing, diaper changing, and bathing [7]. Autoinoculation, where individuals transmit the virus to themselves, has also been identified as a possible transmission route, as seen in cases of children with genital warts who have no history of sexual abuse [50].

Still, cases of anogenital warts in children should raise concern and lead to careful consideration of the possibility of sexual abuse. A systematic review conducted by Awasthi et al. found a significant association between anogenital warts in children over the age of two and increased odds of sexual abuse. Genital wart location, unlike perianal location, was a significant predictor of sexual abuse, but HPV typing was not a reliable method for determining abuse [52]. Another study found that the likelihood of detecting HPV increased with the probability of child sexual abuse, even though anogenital warts themselves were observed in only a small portion of the children studied [53].

## 4. HPV Life Cycle and Epithelial Differentiation

**Figure 4:**
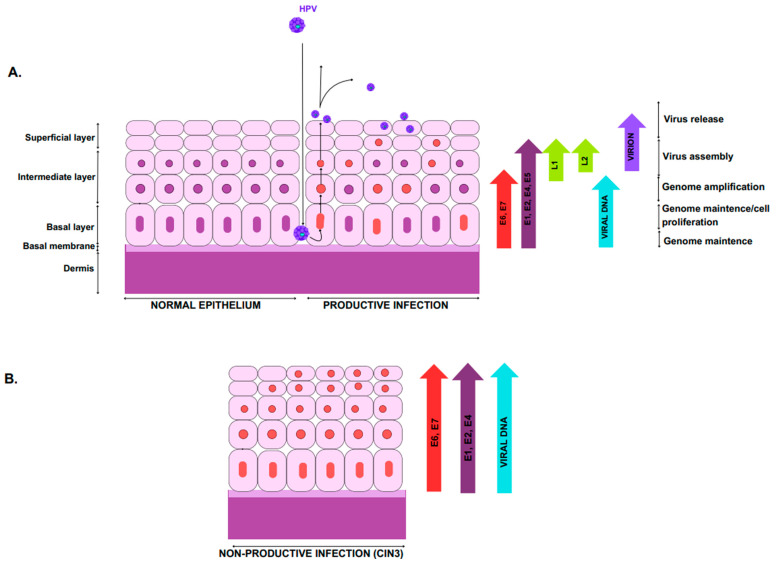
High-risk Alphapapillomavirus life cycle. (**A**) HPV infection begins when the virus gains access to the basal layer of epithelial cells through disruptions in the epithelial barrier. The virus’s gene expression is closely tied to the differentiation of epithelial cells. Early in the infection, cells express E6 and E7, which drive the cell cycle and stimulate division. In the middle layers, proteins essential for viral genome amplification are upregulated, facilitating the process. As cells differentiate, HPV enters the productive phase, leading to the expression of late genes, including L1 and L2, culminating in viral assembly and release. (**B**) However, in HPV-associated neoplasia, late gene expression is delayed, limiting virion production to smaller areas near the epithelial surface. This often results in a non-productive or abortive infection, with deregulated E6/E7 expression facilitating the integration of HPV DNA into the host genome, disrupting normal viral replication and leading to persistent high-level E6/E7 expression, which can contribute to genetic errors in the host.

Regardless of the transmission route, at first, HPV may cause a transient infection that can progress to a persistent one. Persistent HPV may either regress on its own or develop into symptomatic clinical lesions. In infants and children, the virus can often clear naturally. Nevertheless, the hypothesis of a “Trojan horse oncogenic strategy”, referring to children as a reservoir for silent high-risk HPV types, was developed as a field for further research [30]. Many papillomaviruses, typically Beta HPV types, evade immune detection by causing long-term, asymptomatic infections with minimal viral gene expression, resulting in extended periods of virion production and a lower risk of immune clearance. On the other hand, certain Alphapapillomavirus types have developed advanced immune evasion strategies, enabling them to cause persistent warts, such as genital warts, in young adults and common warts in children, even in individuals with healthy immune systems. Productive and non-productive infections differ in the regulation of the Alphapapillomavirus life cycle (Figure 4). The activation of the viral late promoter in upper epithelial layers is essential for increasing viral proteins that are crucial for genome amplification (E1, E2). These cells, often containing high levels of viral proteins, are eventually shed from the epithelial surface. In HPV-associated neoplasia, late gene expression is delayed, and while the sequence of events remains similar, the production of virions is limited to the epithelial surface, leading to non-productive or abortive infection [51]. The clinical implications of these findings emphasise the need for robust screening and vaccination programs, especially in young populations. Further research is required to elucidate the mechanisms behind the immune evasion and persistence of HPV, particularly in children who may serve as a reservoir for oncogenic HPV types. Understanding these mechanisms may pave the way for new therapeutic interventions aimed at enhancing immune responses and reducing the burden of HPV-associated diseases.

## 5. Manifestations of HPV Infection

### 5.1. Skin Warts

Skin warts are the most common HPV manifestation amongst cutaneous HPV types [30]. They affect around 10% of the population, appearing at any age. However, the prevalence has increased, especially among school-aged children between 12 and 16 years old [10]. Warts can be classified as common, flat, and plantar, depending on their morphology and location [54]. Common warts are most associated with HPV 1–4, 7, 10, 26–29, and 57 [10]. They stand for 70% of skin warts [30]. Typically, common warts are multiple, with the mother warts followed by adjacent ones [55]. Flat warts occur on hands, arms, and torsos as flat hyperpigmented papules, giving an impression of tanned skin [56]. Plantar warts tend to appear as single, and multiple warts occupy the soles of the feet. These are known to be painful, contrary to the other types [54]. Several studies report that warts without treatment may spontaneously clear after two years. Thus, a “wait and watch” strategy is sometimes suggested [7]. However, plantar lesions are known to be more resistant to the treatment compared to warts located elsewhere on the skin [54].

### 5.2. Anogenital Warts

Anogenital warts are caused by HPV, typically subtypes 6 and 11, though more than 30 other subtypes can also be responsible for the disease [57]. This type of warts in children is much less common than in adults. Nonetheless, since 1990, the prevalence has shown an increasing tendency. Anogenital warts in children are a controversial topic as they can be transmitted sexually or non-sexually. Thus, children presenting with this type of wart should be evaluated in the context of sexual abuse. Sexual contact has been earlier suggested to be the most frequent mode of transmission. However, more recent studies suggest other modes, such as vertical and horizontal transmission, to be more likely. The association between HPV infection and sexual abuse is correlated with age and shows an increasing tendency. In children younger than 4 years old, it is advisable to strongly consider the possibility of non-sexual transmission when there are no other STIs, no history of sexual abuse, or other clinical indicators.

Children who are over 4 years old are more likely to be exposed to sexual transmission. Girls are affected more often than boys in a ratio of 3 to 1.7 [14]. In girls, the main locations are vulvar, vaginal, urethral, and perianal areas, whereas boys usually suffer from warts placed in the perianal area with occasional penile warts [30]. The clinical characteristics depend on numerous aspects, but most infections remain subclinical, and the virus is detected in seemingly healthy skin and mucous membranes. They usually appear as skin-coloured papules that can be flat or pedicled and sometimes may even have a cauliflower-like appearance. Typically, they are asymptomatic, but bleeding, pain, or pruritus may occasionally occur [14].

### 5.3. Epidermodysplasia Verruciformis

Epidermodysplasia verruciformis, also known as Lewandowsky–Lutz dysplasia, is a rare cutaneous manifestation of HPV invasion. It is mainly linked to HPV 5 or HPV 8 infection, inheritance, or acquired states of immunodeficiency [58]. Typically, the disease occurs spontaneously, but in 25% of patients, it is linked to X chromosome recessive inheritance [56]. Epidermodysplasia verruciformis is characterised by two types of skin manifestations that primarily appear in childhood. Plaques with a morphology of pityriasis versicolor are localised on the face and trunk, while papule-like lesions tend to occur on extremities. Even in most severe cases, mucous membranes remain clear [56,59]. As this type of HPV infection has a high risk of malignancy, it is crucial to monitor the lesions closely. At least one lesion might become malignant in 50% of affected individuals [56].

### 5.4. Recurrent Respiratory Papillomatosis

RRP is the most common benign neoplasm of the larynx that appears amongst children with a background of infection with HPV 6 or 11 [45,60]. The infection is most likely acquired through perinatal transmission, with higher prevalence amongst first-borns and those delivered vaginally [30]. It is characterised by the repeated emergence of benign papillomas along the epithelial lining of the upper respiratory tract, encompassing the larynx, vocal cords, arytenoids, subglottis, and trachea. The most frequently affected region is the mucocutaneous junction of the true vocal cords. Additionally, extra-laryngeal sites, such as the lungs, oropharynx, oral cavity, and nasal cavity, may also become involved [30]. It might cause complete airway obstruction, and typical symptoms presented by affected children are hoarseness, cough, stridor, weak cry, or dysphonia [61]. The disease might be life-threatening, especially when it results from infection with HPV 11, which is considered to be more aggressive.

### 5.5. Retinoblastoma

Retinoblastoma is the most common intraocular malignancy among children, and if left untreated, it is fatal [62]. Retinoblastoma can be inherited. It is hypothesised that HPV, especially HPV 16 and 18, may also be a risk factor as these types contain pRB-inactivating protein [63]. Several studies have been conducted to evaluate this hypothesis; however, the results were not coherent. As HPV has been detected in samples of retinoblastoma from 0% to 82%, some studies have supported the idea of an association between HPV and retinoblastoma [7,63,64]. In contrast, others question this link due to a lower prevalence of HPV in researched cases than in a normal population [65]. However, the type of method and examined tissue should be taken into account due to its diversity between the investigations, as polymerase chain reaction characterised by the highest sensitivity for HPV DNA was not the only method used and the ophthalmic tissue analysed was not always fresh, which would confer a higher rate of HPV DNA detection [7].

### 5.6. Conjunctival Papilloma

A benign squamous cell tumour of the conjunctiva, caused by HPV 6 and 11, can appear at any age with higher prevalence in the third and fourth decades of life [66]. However, 1–10% of the conjunctival lesions in children and adolescents are caused by conjunctival papilloma. Infants are mainly infected through vaginal delivery while older children as well as adolescents are mostly infected through sexual transmission and direct autoinoculation [7]. In this case, HPV impairs the functioning of the p53 tumour suppressor gene. However, to allow tumour progression, both copies of the p53 gene should be damaged. In the “two-hit” theory, damage is first inflicted by UV radiation, while HPV-associated oncogenesis comprises the second hit [67]. The tumour has a fibrovascular core covered by papillary projections and is more likely to be sessile in adults and pedunculated in children. Individuals affected by conjunctival papilloma should be regularly monitored as they are at higher risk of developing laryngeal papilloma [7].

### 5.7. Cervical SILs

Cervical squamous intraepithelial lesions (SILs) are a kind of precursor of invasive squamous cervical carcinoma; however, progression to cancer is a rare case taking up to 20 years [68]. They can be divided into two groups: low-grade and high-grade SILSs. The vast majority of low-grade SILs fully regress while high-grade SILs are much more likely to progress into cervical cancer. In research conducted amongst paediatric and adolescent patients in the USA, the prevalence of SILs was 3.77%, with no cases of carcinoma, even though 18% of patients presented high-grade SILs [69]. Even though only a small percentage of HSILs progress to cancer and most LSILs regress completely, the persistence of SILs amongst sexually active adolescents is of clinical significance as the SIL-negative population has a far lower risk of developing invasive carcinoma [7].

## 6. Strategies to Prevent HPV Infection and Treat HPV-Related Lesions

### 6.1. Treatments of HPV-Related Lesions

Several therapies have been used to treat HPV-related lesions, with varying degrees of success in paediatric populations (Table 1).

### 6.2. Preventive Vaccination

HPV vaccines are based on virus-like particles (VLPs) that spontaneously self-assemble from pentamers of the L1 major capsid protein of HPV [93]. In order to ensure rapid access to local lymph nodes, HPV L1 VLP vaccines are administered intramuscularly, where dendritic cells recognise and transfer the VLPs. In the lymph node, the immune cascade is triggered, initiating a T-cell-dependent B-cell response. HPV L1 VLP vaccines elicit high levels of neutralising antibodies against L1, and immune memory is established [94].

At the moment (Table 2), there are six licensed prophylactic HPV vaccines, four of which have been prequalified by the WHO: quadrivalent Gardasil^®^ (HPV-6, HPV-11, HPV-16, HPV-18), bivalent Cervarix^®^ (HPV-16, HPV-18), nonavalent Gardasil 9^®^ (HPV-6, HPV-11, HPV-16, HPV-18, HPV-31, HPV-33, HPV-45, HPV-52, HPV-58), and bivalent Cecolin^®^ (HPV-16, HPV-18). Other vaccines, not yet prequalified by the WHO, are the bivalent Walvax recombinant HPV vaccine (HPV-16, HPV-18), which is currently under review by the WHO, and quadrivalent Cervavax (HPV-6, HPV-11, HPV-16, HPV-18), which has been licensed nationally in India [95,96,97].

It is now recommended by the WHO that girls aged 9–14 as well as 15–20 receive one- or two-dose schedules and women over 21 receive two doses with an interval of 6 months. Each vaccine manufacturer has proposed various vaccination schedules for their products as well as dosing variants dependent on the age of the recipient. For recipients before the age of 15, schedules composed of two doses are proposed, whereas for recipients 15 years old or older, two doses are proposed in vaccination schedules [98]. Furthermore, the Centers for Disease Control and Prevention (CDC) recommend three doses of the HPV vaccine for individuals starting vaccination on or after their 15th birthday, as well as for immunocompromised individuals. The third dose should be administered 6 months after the first dose (0, 1–2, 6 months schedule) [99]. Although there are slight differences in immune response at different ages, such as higher antibody production in younger adolescents compared to older adolescents and adults (who still exhibit an effective immune response), the recommended age for vaccination is primarily based on the typical age of sexual initiation. That is why the WHO recommends the HPV vaccine for girls before the age of 14 [100].

All HPV vaccines demonstrated very high levels of efficacy in preclinical trials on large groups against their corresponding vaccine-targeted CIN2+ lesions. Gardasil^®^ showed 98% efficacy, Cervarix^®^ exhibited 92.9% efficacy in one study and 89.5% in another, Gardasil^®^ 9 showed 97.1% efficacy, and Cecolin^®^ demonstrated 100% efficacy [97].

The adverse effects of HPV vaccination are most often reported to be mild and transient and include pain and swelling at the injection site, headache, fever, and vomiting [101]. The majority of adverse effects occur after the first dose. Serious adverse effects have been reported in 0.1% of vaccine recipients [102].

The length of protection provided by a new vaccine cannot be determined at the current stage of its introduction, although some research shows that after 5–6 or 4 years, the efficacy remains greater than 98% [94]. Studies have shown that the risk of subsequent CIN2+ can be reduced by 43–67% by peri-treatment vaccination with 2vHPV or 4vHPV vaccines [103]. The routine vaccination of boys should also be considered. This approach would help prevent diseases caused by HPV in men and protect their sexual partners from HPV infections, bringing populations closer to herd immunity. The vaccination of these groups should be considered in countries where the cost and availability of HPV vaccines permit it. Vaccination and screening protocols should be tailored to individual populations based on a cost–benefit–risk analysis [98].

The results of the meta-analysis suggest that a single-dose HPV vaccine could demonstrate similar efficacy to two- or three-dose schedules [104]. Antibody seropositivity has been reported to be high in recipients of any number of doses of HPV vaccine, and antibody levels have been reported to remain stable for at least 11 years after receiving a single dose. However, recipients of one dose tend to have lower antibody levels compared to those who received two or three doses [105]. Implementing a single-dose HPV vaccine could increase accessibility and present a promising solution, especially for underserved populations. Therefore, further research in this area is essential [101]. There is evidence showing that many children are exposed to HPV before sexual initiation, even at the age of 10 [106]. In immunosuppressed children, vaccination against HPV should be seriously considered. These children more often suffer from HPV complications and are up to 100 times more likely to develop HPV- associated malignancies [107]. Children with cancer, HIV infection, haematopoietic stem cell transplantation, and rheumatologic diseases are recommended an HPV vaccine and booster doses [108]. Also, three-dose schedules are recommended for these patients instead of the standard two-dose schedule [107]. One study examined the response to a three-dose quadrivalent HPV vaccination in immunosuppressed children, and HPV was immunogenic in all patients, with no significant adverse events observed [106]. There has been a lot of discussion surrounding therapeutic HPV vaccines. New (genetic, protein-based, peptide-based, dendritic-based) vaccines are being developed to enhance cellular immunity [109]. Adjuvant improvements of existing therapeutic vaccines might increase their effectiveness as well. Furthermore, despite the high costs of implementation and other difficulties, personalised therapeutic vaccines have been suggested. The concept involves the next-generation sequencing of novel epitopes of self-antigens that arise due to mutations in tumour DNA [110]. There have been reports of recent advancements in the development of mRNA vaccine types, which have shown promising results in mouse models targeting HPV-related malignancies. Three vaccines that result in the production of gDE7 protein through translation have been developed. Additionally, a personalised mRNA vaccine has been developed and tested in clinical trials on patients, with promising results. Although the instability of mRNA vaccines presents a problem, innovations are being made to tackle it [111].

## 7. Conclusions

HPV infections are widespread among children and adolescents, manifesting in conditions ranging from common skin warts to more severe issues, such as anogenital warts, epidermodysplasia verruciformis, and RRP.

Management strategies include preventive measures, such as HPV vaccination, which is crucial for reducing HPV-related diseases, as well as various treatments, including topical agents, cryotherapy, lasers, and photodynamic therapy for more severe cases.

Advances in vaccine technology, including mRNA vaccines, hold promise for enhanced HPV prevention and treatment. Integrating effective vaccination with targeted therapeutic approaches is key to improving health outcomes and advancing public health initiatives.

## Figures and Tables

**Table 1:** Treatments for HPV-related lesions.

Method	Indications for Use	Age Requirement	Limitations	Efficacy	References
**Procedures**					
Cryotherapy	Cutaneous warts	Patients > 2 years old were treated; may be used in children <2 years old, but it is generally not the preferred treatment	Lower clearance rates than other treatments (CPS formulation, immunotherapy, laser, topical antivirals, intralesional bleomycin). Possible side effects: pain, blistering, scarring, and hyperpigmentation or hypopigmentation; contraindicated for patients with cryoglobulinemia, multiple myeloma, Raynaud disease, cold urticaria, and disordered peripheral circulation.	45–75%; cryotherapy is more effective for nonplantar warts, yet no clinically significant differences were seen after 13 weeks	[70,71,72,73]
Surgical curettage	Resistant and widespread cutaneous warts		Possible side effects: pain.	55.6% over 2 years	[70]
Electrocoagulation	Filiform or small isolated warts	Patients > 3 years old were treated	Not a mainstay treatment for children; it requires the use of local anaesthesia and can leave a scar.		[71]
CO_2_ laser	Palmar–plantar warts; can be a first-line treatment for periungual and subungual warts	Patients > 2 years old were treated	Possible side effects: painful and can cause scarring. Pulsed-dye lasers can cause pain and scarring but less than CO_2_ lasers.	Complete resolution rates of 75% for children with palmar–plantar warts	[71,74]
Photodynamic therapy	Refractory palmar and plantar warts		Possible side effects: erythema, a burning sensation, and pain. Procedure is painful; it is not recommended in children and is a high-cost treatment	Cure rate of 56% for treated warts after 18 weeks of treatment	[71,75]
**Topical**					
Salicylic acid	Cutaneous warts; first-line treatment can be combined with 5-fluorouracil, lactic acid, cryotherapy, imiquimod, and cantharidin	It is not used in children under 2 years of age	Slow response (2–3 months). Possible side effects: skin irritation, pain, bleeding, and salicylic acid toxicity (tinnitus, nausea and vomiting).	Approximately two-thirds of all warts clear within 3 to 6 months of treatment with topical salicylic acid	[70,71,76,77]
Lactic acid	Cutaneous warts; combined with salicylic acid	It is not used in children under 2 years of age	Possible side effects: skin irritation.	Clearance rate of 58.8–75% for SA+ lactic acid combined therapy	[70,78]
Tretinoin	Common warts, resistant flat warts	Patients ≥ 12 years old (FDA)	FDA off-label use. Possible side effects: irritant contact dermatitis.	Clearance rate of 85% in children treated with tretinoin 0.05%	[79,80,81,82]
5-fluorouracil	Palmoplantar warts; combined with salicylic acid	Not used in children under 6 years old (ChPL); inability to use in infants	Possible side effects: inflammation, skin erosion, and hyperpigmentation or hypopigmentation	Clearance rate of 20% for SA+ 5-fluorouracil combined therapy	[70,78]
Imiquimod	Common warts, periungual and subungual warts, anogenital warts	Not FDA-approved for children under 12 years old but was effective in 6-month-old children	Possible side effects: local erythema, itch, vesiculation, ulceration and burning sensation, low pain.	Complete resolution in 80% of patients	[71,83,84,85]
Bleomycin	Cutaneous warts	Not established (FDA)	Possible side effects: eschar formation and blackening of the skin.	Broadly varying cure rates (16–94%)	[86]
Cantharidin	Cutaneous warts	FDA-approved for patients ≥ 2 years old	Possible side effects: burning, erythema, pain, and pruritus.	96% cure rate	[87,88]
Cidofovir	Warts on the oral mucosa, hands, and anogenital region	Patients ≥ 9 years old were treated in a trial	Possible side effects: pain, pruritus, and rash at the application site.	Clearance in 47%; more studies are required to further estimate the efficacy	[87,89]
**Intralesional**					
Bleomycin	Common warts, plantar warts	Not widely studied in paediatric population; not recommended for young children	Possible side effects: pain, dyspigmentation, necrosis; due to high pain levels, an anaesthetic may be necessary.	95 to 97% resolution rate	[71,75,90]
5-fluorouracil	Palmoplantar warts, anogenital warts	Not FDA-approved for paediatric patients with anogenital warts	Possible side effects: inflammation, blistering, pain, skin erosion, and hyperpigmentation or hypopigmentation	Studies have shown it to be effective, but it is not FDA-approved for children	[14,71,78]
Candida albicans antigen	Common warts	Patients ≥ 5 years old were treated in a trial	Possible side effects: pain, subsequent bullae, oedema, desquamation, fever, burning, and blistering.	Complete resolution without recurrence in 72%	[76,91]
Cidofovir	Common warts	Patients > 3 years old were treated in a trial	It is avoided as it is associated with nephrotoxicity, ocular injury (anterior uveitis, retinal detachment, iritis and permanent loss of vision), and teratogenicity. Possible side effects: pain, burning sensation, itching, erythema, and post-inflammatory hyperpigmentation.	Clearance in 99%; more studies are required to further estimate the efficacy	[87]
MMR vaccine	Refractory cutaneous warts on the head and neck region and limbs; periungual and subungual warts	Patients ≥ 11 years old were treated in a trial	No response in 33% of patients in the trial. Possible side effects: mild pain during injection.	At 6 months of follow-up: full resolution of warts in 62.8% and partial response in 4.2% of patients	[92]

**Table 2:** Overview of licensed and prequalified HPV vaccines.

Vaccine (Manufacturer)	Valence, HPV Types	Adjuvant	Registration Year	Vaccine Administration	Vaccine Schedule
Gardasil^®^ (Merck & Co., Rahway, NJ, USA)	Quadrivalent (6, 11, 16, 18)	Amorphous aluminium hydroxyphosphate sulphate	2006	I.M.	9–14 years: 0, 6 months; from the age of 15: 0, 2, 6 months
Cervarix^®^ (GlaxoSmithKline, Tsim Sha Tsui, Hong Kong)	Bivalent (16, 18)	ASO4: 3-O-deacylo-4′-monofosforylolipid A (MPL), adsorbed on aluminium hydroxide	2007	I.M.	9–14 years: 0, 6 months; from the age of 15: 0, 1, 6 months
Gardasil9^®^ (Merck & Co.)	Nonavalent (6, 11, 16, 18, 31, 33, 45, 52, 58)	Amorphous aluminium hydroxyphosphate sulphate	2014	I.M.	9–14 years: 0, 6 months; from the age of 15: 0, 2, 6 months
Cecolin^®^ (Xiamen Innovax Biotechnology, Xiamen, China)	Bivalent (16, 18)	Aluminium hydroxide	2020	I.M.	9–14 years: 0, 6 or 0, 1, 6 months; from the age of 15: 0, 1, 6 months
Walvax recombinant HPV vaccine (Hanghai Zerun Biotechnology, Shanghai, China; Subsidiary of Walvax Biotechnology, Shanghai, China)	Bivalent (16, 18)	Aluminium phosphate	2022	I.M.	9–14 years: 0, 6 or 0, 2, 6 months; from the age of 15: 0, 2, 6 months
Cervavac^®^ (Serum Institute of India, Pune, India)	Quadrivalent (6, 11, 16, 18)	Aluminium (Al3+)	2022	I.M.	9–14 years: 0, 6 months; from the age of 15: 0, 2, 6 months
